# Epitranscriptomic control of telomere maintenance

**DOI:** 10.1007/s11033-026-11699-w

**Published:** 2026-03-19

**Authors:** Ehsan Pashay Ahi, Zeinab Ghasemishahrestani

**Affiliations:** 1 Organismal and Evolutionary Biology Research Programme, Faculty of Biological and Environmental Sciences, University of Helsinki, Viikinkaari 9, Helsinki, 00014 Finland; 2 Faculty of Medicine, University of Turku, Kiinamyllynkatu 10, FI-20520, Turku, Finland; 3 MRC-SA Wound Healing & Keloid Research Unit, Division of Dermatology, Department of Medicine, Groote Schuur Hospital, University of Cape Town, Cape Town, South Africa

**Keywords:** Epitranscriptomics, Telomere maintenance, Telomerase ribonucleoprotein, Telomeric repeat-containing RNA, Alternative lengthening of telomeres, RNA:DNA hybrids, RNA modifications

## Abstract

Telomere maintenance has been portrayed primarily as a problem of DNA–protein architecture and chromatin control, yet a complementary layer has been revealed at the level of RNA chemistry. In this Review, RNA modifications and their writer–reader–eraser and RNA-editing systems are integrated into a framework for chromosome-end homeostasis. Epitranscriptomic regulation of the telomerase ribonucleoprotein is examined, and assembly, activity, and recruitment are shown to be reshaped by chemical marks on TERC, specialized RNA capping, and processing pathways. Telomeric transcripts, particularly TERRA, are discussed as modified substrates whose stability, trafficking, and propensity for telomeric RNA: DNA hybrid formation can be tuned by RNA marks and their readers. Downstream consequences for replication stress, DNA damage signaling, and recombination-driven alternative lengthening of telomeres are summarized, together with emerging examples in which modification of telomere-factor mRNAs has been linked to rewiring of maintenance networks. Across these themes, links to telomeropathies, aging-associated inflammation, environmental stressors, and cancer are collated to connect mechanism to phenotype. Experimental bottlenecks and opportunities—site-resolved mapping, locus-targeted editing, and pharmacologic modulation of RNA-modifying enzymes—are outlined as routes toward causal models and therapeutic utilization.

## Introduction

Telomeres are specialized nucleoprotein structures that cap chromosome ends and are required to prevent end-to-end fusions, illegitimate repair, and progressive genome instability [1, 2]. Progressive telomere attrition is typically imposed by incomplete end replication and nucleolytic processing, and critically short or perturbed telomeres are sensed as DNA lesions that can activate checkpoint signaling and durable proliferative arrest or cell death [1]. Telomere dysfunction has consequently been positioned as a causal contributor to degenerative phenotypes and as a constraint on malignant evolution, while also being recognized as the basis of inherited telomere biology disorders (telomeropathies) in which tissue failure arises from insufficient telomere reserve [1]. Physiologic telomere maintenance in humans is mediated primarily by telomerase, a ribonucleoprotein enzyme whose core includes the reverse transcriptase TERT and the template RNA TERC (hTR), and whose activity is restricted to specific developmental stages and cell types [3]. In contrast, long-term proliferation in cancer is commonly supported by reactivation of telomerase or by engagement of a telomerase-independent recombination-based program termed alternative lengthening of telomeres (ALT) [4]. ALT was originally evidenced in subsets of tumors and tumor-derived cell lines as telomerase-negative states associated with long, heterogeneous telomeres, establishing telomere maintenance as a pluralistic problem rather than a telomerase-only phenotype [5]. A mechanistic and clinical distinction between telomerase-positive and ALT-positive tumors has since been emphasized, both because diagnostic strategies differ and because therapeutic vulnerabilities appear to be shaped by the telomere maintenance mechanism (TMM) that is selected [4].

Although telomere maintenance has long been narrated through the lens of DNA replication, recombination, and telomeric chromatin organization, an additional RNA layer has been embedded in the process [3]. Emerging models of human telomere regulation likewise emphasize that telomeric chromatin state and telomeric transcription are tightly coupled features of chromosome-end homeostasis [6]. Telomerase itself is assembled, trafficked, and regulated through RNA-centered biogenesis steps that determine whether catalysis and telomere recruitment are achieved [3]. In parallel, telomeric repeat-containing RNA (*TERR*A) is transcribed from subtelomeric regions and has been implicated in telomere homeostasis through effects on telomeric chromatin, transcription–replication conflicts, and the formation and processing of telomeric RNA: DNA hybrids (R-loops) [7–9]. Recent promoter-level analysis using the T2T-CHM13v2.0 human reference genome further refined this view by identifying TERRA promoters at 39 of 46 subtelomeres and revealing numerous intrachromosomal TERRA-like promoters, underscoring that the TERRA pool may arise from a broader and more heterogeneous set of loci than previously appreciated [10]. Importantly, telomere-to-telomere heterogeneity in *TERRA* abundance and composition has been highlighted, implying that telomeric RNAs are not merely bulk by-products but locus-tuned regulators whose regulation can be mechanistically instructive for telomere stability [7]. RNA-centered regulation of chromosome ends is not restricted to mammals. In budding yeast, telomerase RNA biogenesis depends on regulated processing and cap maturation, and short telomeres accumulate TERRA and telomeric RNA: DNA hybrids that can promote recombination-based rescue [11]. Comparative work in fungi has also shown that telomerase RNA architecture is more deeply conserved than primary sequence alone would suggest [12]. In plants, identification of bona fide telomerase RNAs and their associated factors has further revealed both conserved and lineage-specific features of telomerase RNP assembly [13].

A further regulatory layer has been introduced by the recognition that RNA molecules are decorated with chemical modifications that can reshape RNA folding, stability, localization, and protein binding without altering primary sequence [14]. The collective landscape of these marks has been described as the epitranscriptome and has been approached through an expanding toolkit that ranges from antibody-based enrichment and chemical conversion to direct RNA sequencing and locus-resolved validation strategies [15–17]. The field has been accelerated by improvements in modification mapping, by the cataloguing of regulatory “writers,” “readers,” and “erasers,” and by the emergence of programmable approaches for engineering individual marks to test causality rather than correlation [14, 18]. In parallel, RNA modifications have been integrated into genome integrity frameworks, where modified RNAs and RNA: DNA hybrids have been shown to influence DNA damage responses and repair outcomes under physiological and stress conditions [19–21]. Given that telomeres behave as endogenous “difficult-to-replicate” loci and can phenocopy persistent DNA lesions when compromised, telomere maintenance has been positioned as a natural arena in which RNA modification–dependent regulation might be especially consequential [1, 19].

Direct links between epitranscriptomic regulation and telomere maintenance have now been demonstrated most clearly in mammalian telomere-defining RNA substrates. A methylated-cytosine mark on human TERC (m⁵C at C106) has been connected to telomerase assembly and catalytic output through an RNA-binding protein–dependent mechanism, and disease-associated TERC variants have been shown to disrupt this modification axis in parallel with telomere shortening phenotypes [22]. On the telomeric transcript side, N⁶-methyladenosine (m⁶A) has been detected on subtelomeric regions of TERRA, and stabilization of TERRA through METTL3-dependent writing and YTHDC1-dependent reading has been linked to telomere protection, telomeric R-loop biology, and homologous recombination features required for ALT [23]. A complementary mechanistic angle has been provided in which METTL3-dependent m⁶A on TERRA has been connected to telomere targeting through m⁶A-facilitated R-loop formation, with downstream telomere damage being induced upon disruption of this pathway and with therapeutic relevance being suggested in ALT-positive neuroblastoma models exposed to METTL3 inhibition [24]. These findings collectively indicate that telomere maintenance can be tuned not only by DNA- and chromatin-level mechanisms but also by RNA chemistry acting on telomerase RNA, telomeric transcripts, and telomere-localized RNA structures.

**Fig. 1:**
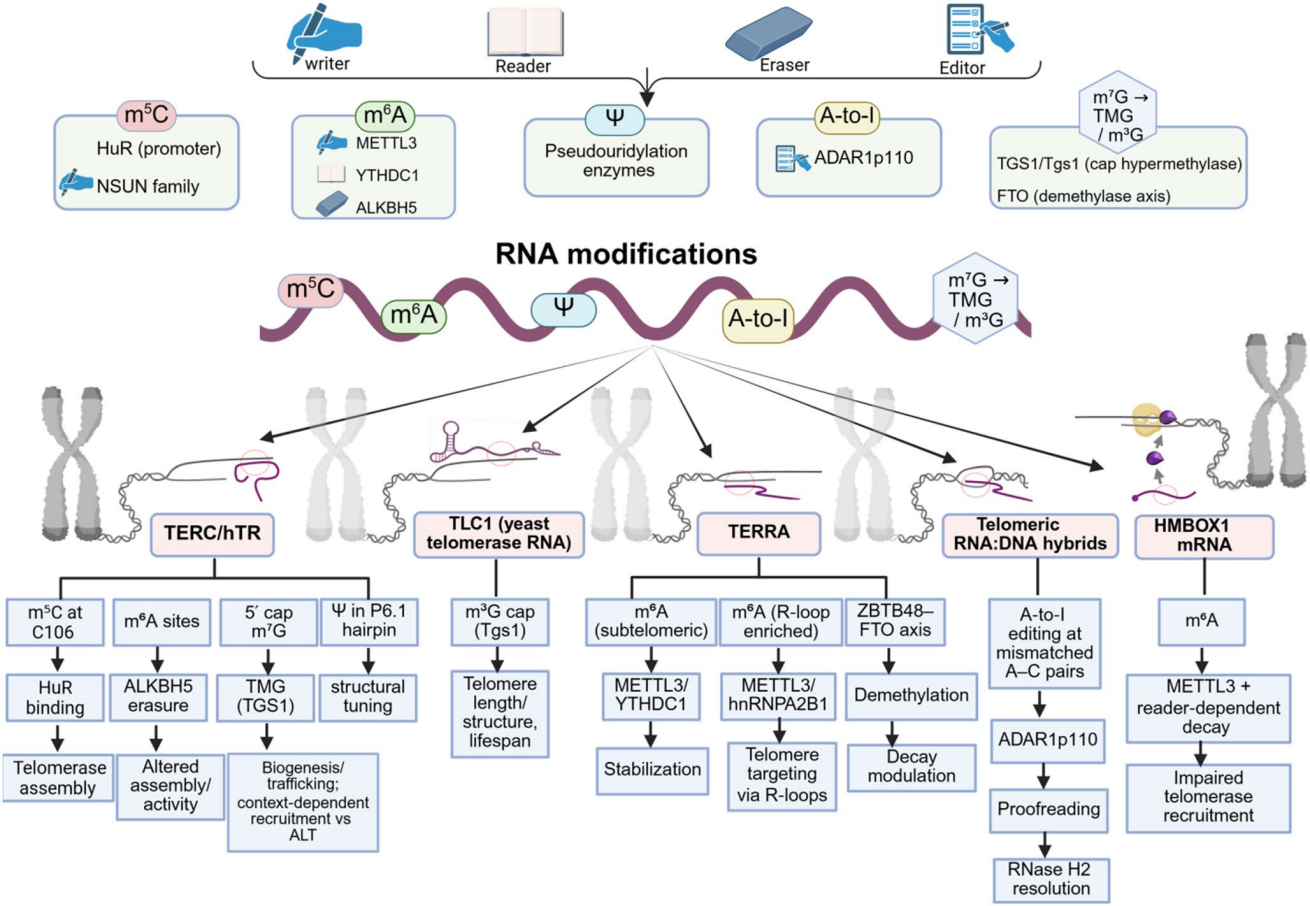
Epitranscriptomic regulation of telomere maintenance. Schematic representation of RNA chemical modifications and their regulatory enzymes illustrating their coordinated roles in telomere biology. The diagram summarizes the distribution and functional consequences of RNA modifications on human telomerase RNA (hTR/TERC), telomeric repeat-containing RNA (TERRA), yeast telomerase RNA (TLC1), telomeric RNA: DNA hybrids (R-loops), and *HMBOX1* mRNA. RNA modifications depicted include N⁶-methyladenosine (m⁶A), 5-methylcytosine (m⁵C), pseudouridine (Ψ), adenosine-to-inosine (A-to-I) editing, and specialized cap structures (m⁷G to TMG; m³G). The corresponding regulatory factors are shown: METTL3 (m⁶A methyltransferase), YTHDC1 (m⁶A reader), ALKBH5 (m⁶A demethylase), HuR (m⁵C-associated stabilizing factor), TGS1/Tgs1 (cap hypermethylase), ADAR1p110 (A-to-I RNA editor), hnRNPA2B1 (m⁶A-dependent RNA-binding protein), and the ZBTB48–FTO demethylase axis. Functionally, m⁵C at C106 of hTR enhances HuR binding and promotes telomerase ribonucleoprotein (RNP) assembly, while m⁶A dynamically regulates RNP formation through ALKBH5-mediated demethylation. TGS1-dependent trimethylguanosine (TMG) capping governs telomerase biogenesis and recruitment, influencing the balance between canonical telomerase activity and alternative lengthening of telomeres (ALT). Pseudouridylation within the P6.1 domain modulates RNA secondary structure and catalytic efficiency.In yeast TLC1, m³G cap hypermethylation is linked to telomere length homeostasis, structural stability, and replicative lifespan. In TERRA, METTL3-mediated m⁶A deposition and YTHDC1 recognition enhance transcript stability, whereas m⁶A enrichment within R-loop fractions promotes telomeric targeting via hnRNPA2B1. The ZBTB48–FTO axis regulates m⁶A demethylation and transcript turnover. Within telomeric RNA: DNA hybrids, ADAR1p110-mediated A-to-I editing facilitates mismatch correction and RNase H2–dependent R-loop resolution. In *HMBOX1* mRNA, m⁶A-dependent destabilization reduces telomerase recruitment efficiency

**Fig. 2:**
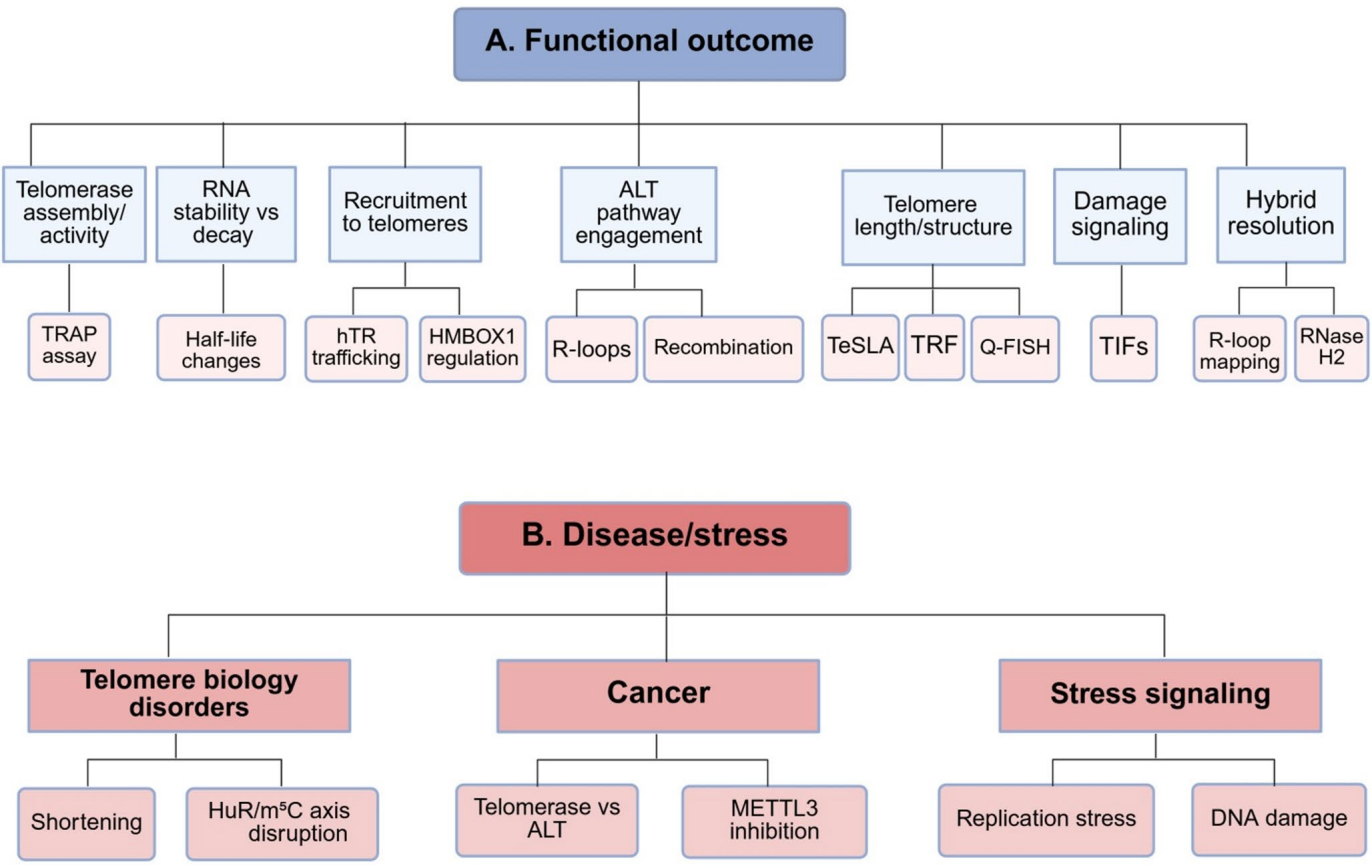
Functional and pathological consequences of epitranscriptomic regulation at telomeres. (**A**) Functional outcomes. Schematic representation of the downstream effects of RNA modifications on telomere homeostasis, including transcript stabilization and degradation, telomerase recruitment, recombination dynamics, regulation of telomere length, activation of telomere dysfunction–induced foci (TIFs), and alternative lengthening of telomeres (ALT) activity, as reflected by C-circle formation and R-loop accumulation. (**B**) Disease contexts. Overview of pathological settings associated with dysregulated telomeric epitranscriptomics, including telomere biology disorders, cancer subtypes utilizing telomerase versus ALT mechanisms, and stress signaling pathways such as replication stress and DNA damage responses

A synthesis focused specifically on “epitranscriptomic control of telomere maintenance” is therefore warranted, because the relevant evidence has been dispersed across traditionally separate literatures: telomerase biogenesis and recruitment, telomeric RNA biology, ALT recombination, and RNA modification–dependent genome stability [3, 4, 7, 14, 15, 19]. In this Review, a framework is provided in which telomere maintenance is treated as an RNA-modifiable system, and the major mechanistic entry points—telomerase ribonucleoprotein regulation, telomeric RNA fate and localization, telomeric R-loop homeostasis, and ALT-associated recombination—are integrated with disease and stress contexts in which telomere phenotypes are most sharply revealed. Operational criteria for causal claims in this area and a unifying framework for how RNA chemistry can influence telomere maintenance states are outlined in Table 1. Key RNA chemical marks and editing events reported to act on telomere-relevant RNA substrates, together with their mechanistic and telomere-maintenance outcomes, are summarized in Table 2 and are schematically integrated in Figs. 1 and 2, which provide an overview of the relevant RNA modifications, their regulatory enzymes, and their downstream effects on telomerase, TERRA, telomeric RNA: DNA hybrids, and chromosome-end homeostasis. It is important to note that, in order to preserve the focus and concision, we have organized it primarily around mammalian examples, which currently provide the most direct mechanistic evidence for telomere-specific epitranscriptomic regulation, while incorporating selected non-mammalian studies where they illuminate conserved or lineage-specific principles. Across taxa, conservation is currently clearer at the level of RNA-centered telomere regulation than at the level of specific telomere-associated epitranscriptomic pathways, which remain more unevenly resolved.

**Table 1:** Building causal models for RNA marks at telomeres: evidence standards and a unifying framework

Section	Item	Details
A. Evidence checklist for epitranscriptomic control	Define the substrate and mark	Specify the RNA (hTR/TERC, TERRA, relevant mRNA, hybrid-associated RNA) and the modification/editing event; ideally include site/region and, where possible, stoichiometry/occupancy.
	Demonstrate directionality	Show that perturbing the writer/reader/eraser/editor changes the mark on the relevant substrate, not only global methylation levels.
	Link to telomere-specific outcomes	Pair perturbations with telomere readouts that distinguish telomerase catalytic output, telomere length distributions, telomere damage (TIFs), ALT markers (e.g., C-circles), and telomeric R-loops.
	Use targeted editing where feasible	Use site-directed install/erase/edit approaches (e.g., Cas13-based) to establish causality rather than correlation.
	Control for indirect stress effects	Monitor replication stress, global DDR, and cell-cycle effects alongside telomere endpoints to avoid mistaking pleiotropy for a telomere-specific mechanism.
B. Unifying framework	Routing codes	RNA marks/caps bias RNA localization and RNP engagement (e.g., hTR cap state; TERRA telomere targeting).
	Hybrid competence signals	Marks/editing tune formation, stability, and resolution of telomeric RNA: DNA hybrids (e.g., m⁶A on TERRA; ADAR1 editing at variant repeats).
	Network rewiring inputs	Marks on telomere-factor mRNAs reshape pathway state indirectly (e.g., HMBOX1 mRNA destabilization impacting telomerase recruitment).

Practical criteria to support claims of epitranscriptomic control of telomere maintenance, and a compact model for how RNA chemical states can influence telomere pathway choice

**Table 2:** Epitranscriptomic modifications on telomere-relevant RNAs and their functional outputs

RNA substrate	Modification/RNA chemistry	Site/region (if defined)	Enzymes/factors	Reported mechanistic effect	Telomere maintenance outcome/context	Key References
hTR/TERC	m⁵C	C106	HuR (promotes axis; methyltransferase not fully defined)	Enhances hTR–TERT assembly and telomerase activity	Telomerase-dependent maintenance; TBD-linked variants disrupt axis	[22, 47]
hTR/TERC	m⁶A	A111 and A435	METTL3 (writer); YTHDC1 (reader); ALKBH5 (eraser)	Alters telomerase assembly/activity; promotion of TERT–TERC assembly	Telomerase regulation; network-state coupling	[3, 50, 56]
hTR/TERC	5′ cap: m⁷G → TMG	5′ end	TGS1 (cap hypermethylase)	Routes hTR biogenesis/trafficking; context-dependent effects on abundance vs. recruitment	Telomerase biogenesis and recruitment; context-dependent constraint	[51–53]
TLC1	m³G cap	5′ end	Tgs1	Links cap hypermethylation to telomere length/structure & lifespan	Evolutionary conservation of cap-based regulation	[54]
hTR/TERC	Pseudouridine (Ψ)	P6.1 hairpin (predicted sites)	Pseudouridylation (installers not resolved in vivo here)	Tunes RNA structure/stability; modest activity/processivity effects in vitro	Catalytic tuning (evidence strongest in vitro)	[55]
TERRA	m⁶A	Predominantly subtelomeric segments	METTL3 (writer); YTHDC1 (reader)	Stabilizes TERRA; prolongs half-life	Telomere protection and ALT-linked phenotypes	[23]
TERRA	m⁶A	R-loop–enriched TERRA fraction	METTL3; hnRNPA2B1 (m⁶A-dependent recruitment)	Promotes telomere targeting via m⁶A-facilitated R-loop formation	ALT-positive neuroblastoma vulnerability; telomeric damage upon disruption	[24]
Telomeric RNA: DNA hybrids	A-to-I editing	Mismatched A–C pairs in hybrids	ADAR1p110 (editor); RNase H2 resolution	“Proofreads” mismatches to enable hybrid resolution	Supports proliferation of telomerase-reactivated cells with variant repeats	[80]
HMBOX1 mRNA	m⁶A	Noted as functional m⁶A target	METTL3; reader-dependent decay pathway	Destabilizes HMBOX1 mRNA → impaired telomerase recruitment to telomeres	Network rewiring: telomere dysfunction/instability in cancer	[95]
TERRA + other RNAs	m⁶A/m⁶Am demethylation targeting	Not site-resolved for TERRA	ZBTB48–FTO axis	Steers demethylase access; modulates decay rates of FTO targets	Candidate telomere-factor control of methylation dynamics	[66]

Summary of RNA chemical marks (and/or RNA editing) directly linked to telomerase regulation, TERRA fate, telomeric R-loop control, ALT features, or telomere-network rewiring. Sites/regions are given where defined; Context indicates telomerase vs. ALT emphasis

## Epitranscriptomic mechanisms and readouts in telomere biology

Epitranscriptomic regulation has been defined as the modulation of RNA fate and function through chemical modifications that alter RNA–protein interactions, RNA structure, and RNA turnover without changing nucleotide sequence [14]. Among the best-characterized internal marks, N⁶-methyladenosine (m⁶A), 5-methylcytosine (m⁵C), pseudouridine (Ψ), and adenosine-to-inosine (A-to-I) editing have been emphasized because well-defined writer enzymes (and, where established, reader proteins and/or erasers) and measurable phenotypes have been established across diverse RNA classes [14, 19, 25]. In addition, cap-adjacent modifications and specialized RNA cap chemistry have been placed within the operational definition of epitranscriptomic control when changes in capping state have been shown to redirect RNA stability, trafficking, and translation [14, 15]. Because telomere maintenance is executed by RNA-containing machines (telomerase) and is strongly influenced by telomere-associated transcripts (notably telomeric long noncoding RNAs), the telomere field has been positioned as a natural setting in which RNA chemistry can exert causal control [14, 19].

For the purposes of this Review, epitranscriptomic control of telomere maintenance has been treated as an inference that should be supported by two evidence layers: (i) RNA modification states (or their regulatory enzymes) should be perturbed in a manner that is definable at the level of specific marks, sites, or substrates; and (ii) telomere maintenance outcomes should be measured using assays that distinguish catalytic telomerase output, telomere length homeostasis, telomere damage signaling, and recombination-based alternative lengthening of telomeres (ALT) [14, 15, 19]. Particular caution has been warranted because RNA-modifying enzymes frequently act on large transcript sets and because telomere phenotypes can be secondary to global changes in proliferation, replication stress, or DNA damage responses [14, 19]. Accordingly, orthogonal validation strategies—ideally combining modification mapping, targeted editing, and telomere-specific phenotyping—have been recommended for causal interpretation [14, 15, 19]. A practical menu of telomere maintenance readouts and epitranscriptomic mapping/editing approaches that support causal inference is provided in Table 3.

**Table 3:** Telomere-maintenance readouts and epitranscriptomic interrogation tools

Question	Assay/approach	What it measures	Strengths	Key pitfalls/controls	Key References
Is telomerase enzymatically active?	TRAP	Telomerase extension activity (PCR readout)	Sensitive; high-throughput	PCR artifacts; normalization; extract quality	[34]
Is telomere length changing (mean)?	qPCR	Relative telomere content	Fast; low input	Provides averages; batch effects	[35]
Is the length distribution changing?	TRF	Size distribution of terminal fragments	Distribution-level insight	Includes subtelomeric DNA; requires DNA quality	[35]
Are shortest telomeres changing?	TeSLA	Shortest-telomere distribution	Sensitive to rare critically short ends	Technical complexity; careful controls	[36]
High-resolution telomere distributions?	Long-read telomere measurement	Digital distributions, structure	Resolves distribution features	Platform/analysis considerations	[37]
Is there telomere-specific DNA damage?	TIF assay	DDR foci colocalizing with telomeres	Telomere-specificity checkpoint	Needs robust colocalization thresholds	[38]
Is ALT active?	C-circle assay	ALT hallmark DNA circles	Quantitative and responsive	Exonuclease controls; sample handling	[39, 40]
ALT in clinical samples?	C-circle (FFPE-adapted)	ALT activity in FFPE tissue	Translational applicability	DNA quality variability in FFPE	[41]
Are telomeric R-loops present/altered?	R-loop mapping (S9.6 DRIP/RNase H1 capture)	Hybrid abundance/landscape	Mechanistic intermediate readout	Method-dependent bias; orthogonal validation	[42, 43]
Where are RNA modifications (global)?	Antibody/chemical mapping; site-resolved methods	Transcriptome-wide mark locations	Discovery-scale	Antibody specificity; stoichiometry ambiguity	[15, 26]
Can we detect marks on native RNAs?	Nanopore direct RNA + mod calling	Native RNA + modification-aware signal	Preserves RNA; isoforms + marks	Signal models; coverage/stoichiometry limitations	[27, 28, 44]
Can we test causality at a specific site?	dCas13 writer/eraser editing	Targeted installation/removal of m⁶A	Causal tests, site-level	Off-targets; guide design; timing	[31–33]
Can we link TERRA methylation to stability?	Targeted demethylation of mapped TERRA site	Local m⁶A change + RNA abundance	Strong causal chain	Requires site-defined target and controls	[23]

Practical menu of assays pairing telomere phenotyping (telomerase activity, telomere length distributions, ALT markers, telomeric damage, telomeric R-loops) with epitranscriptomic mapping/editing strategies suited for causal tests

### Mapping and quantifying RNA modifications in telomere-relevant contexts

A technical gap has historically been imposed by the limited resolution, specificity, and quantitative interpretability of many transcriptome-wide mapping methods [15, 26]. Antibody-enrichment approaches have enabled broad discovery but have often been constrained by epitope cross-reactivity, peak-level resolution, and difficulties in estimating stoichiometry at individual sites [15, 26]. Base-resolved strategies have therefore been increasingly adopted, including antibody-assisted crosslinking methods, enzyme-assisted detection, and chemical conversion schemes, with the goal of supporting site-level mechanistic models rather than correlation-driven annotation [15, 26]. The need for careful controls has been underscored, including matched negative controls, replicate-driven statistics, and validation by independent approaches (for example, targeted assays or mass spectrometry) when quantitative conclusions are drawn [15, 26].

Direct RNA sequencing has been positioned as a complementary route because native RNA molecules can be read without reverse transcription or amplification, thereby preserving information that is otherwise erased during cDNA workflows [26, 27]. Modification calling from nanopore signal has been advanced by comparative frameworks that avoid training sets, by tool benchmarking, and by systematic evaluation of computational pipelines under realistic coverage and stoichiometry conditions [28]. It has also been emphasized that, despite rapid progress, direct RNA modification detection remains sensitive to library preparation biases, basecalling models, and analytical assumptions; clinical translation has consequently been discussed as plausible but still method-limited for many applications [26–28]. Long-read RNA sequencing benchmarks in human cell lines have further reinforced that transcript-isoform complexity and library preparation choices can materially affect downstream interpretation, including modification-aware analyses [29].

### Perturbing RNA modification pathways: from global disruption to programmable editing

Mechanistic dissection has often begun with genetic manipulation of writers, readers, or erasers, or with pharmacologic perturbation where selective inhibitors are available [14, 19]. However, because global perturbations can trigger pleiotropic phenotypes, targeted approaches have been increasingly emphasized to test whether a specific mark at a defined position is sufficient to alter a telomere-relevant pathway [14, 19].

Programmable epitranscriptomic editing has therefore become a pivotal enabling technology. Site-directed installation and erasure of m⁶A have been demonstrated using CRISPR-guided platforms, including dCas9-based systems that use guide RNAs and PAMmer-type auxiliary DNA oligonucleotides to engage RNA substrates, as well as dCas13-based systems that directly target RNA [30, 31]. Cas13-directed writer fusions have been shown to deposit m⁶A at selected RNA sites with measurable downstream effects, providing an operational route from mapping to causality [30]. Temporal control has also been engineered: inducible and reversible m⁶A editing has been achieved through chemically induced proximity and ligand-responsive recruitment, enabling kinetics and reversibility to be tested rather than inferred [32]. More recently, light-inducible editing has been reported to support reversible control with reduced off-target effects, further strengthening the feasibility of causal experiments in dynamic telomere/stress settings where timing can be decisive [33]. Overall, these platforms have been framed as essential for telomere biology because many telomere phenotypes emerge gradually, can be cell-cycle restricted, and can be confounded by selection during long culture windows.

### Telomere maintenance readouts suited to epitranscriptomic studies

Telomerase catalytic output has been most commonly quantified using TRAP-based assays, with extensive guidance having been provided on sample preparation, internal controls, PCR artifacts, and interpretation limits [34, 35]. Because telomerase regulation can be altered without immediate changes in bulk telomere length, TRAP readouts have been regarded as complementary rather than redundant to telomere length assays [34, 35].

Telomere length has been measured by multiple orthogonal approaches that differ in throughput, resolution, and the extent to which distributions (rather than means) can be recovered [35]. Hybridization- and PCR-based assays (including TRF analysis, qPCR, Q-FISH, and Flow-FISH) have been widely used for population-level comparisons and for research and some clinical workflows, while single-molecule and shortest-telomere–focused assays have been highlighted as particularly informative for mechanistic studies of telomere maintenance failure [35]. TeSLA has been introduced as a method designed to quantify the distribution of the shortest telomeres across chromosome ends, thereby enabling phenotypes driven by rare critically short telomeres to be captured [36]. Long-read sequencing–based approaches have further expanded the measurement landscape: digital telomere measurement by nanopore sequencing has been reported to provide high-resolution telomere length distributions and to distinguish healthy aging from telomere maintenance disorders at the level of distribution structure rather than a single summary statistic [37].

Telomere integrity has been operationalized through assays that report DNA damage signaling at telomeres. Telomere dysfunction-induced foci (TIFs) have been quantified by colocalization of telomeric proteins with canonical DNA damage markers, enabling telomere uncapping or damage to be detected even when global DNA damage remains modest [38]. Because RNA modification pathways are deeply intertwined with DNA damage responses and replication stress signaling, TIF measurements have been treated as a critical “specificity checkpoint” when telomere phenotypes are proposed [19, 38].

ALT activity has been distinguished from telomerase-mediated maintenance by a set of molecular and cytologic markers, among which C-circles have been widely treated as a quantitative and responsive hallmark [39, 40]. Protocol-level considerations (including exonuclease controls and the responsiveness of the assay to ALT modulation) have been detailed, supporting reproducible application across experimental settings [39]. Importantly, C-circle detection has also been adapted to clinically relevant specimens, including formalin-fixed paraffin-embedded tissue, thereby enabling ALT activity to be assessed in patient-derived material where epitranscriptomic states may be of translational interest [41].

Finally, telomeric RNA: DNA hybrids (telomeric R-loops) have been increasingly incorporated as mechanistic readouts because telomeric transcripts and RNA-modifying enzymes can converge on hybrid formation, processing, and stability [19, 42]. Genome-wide R-loop mapping approaches have been formalized around S9.6-based immunoprecipitation methods and RNase H1–based capture modalities, with differences in specificity, resolution, and analytical pipelines having been clearly articulated [42]. Meta-analytic quality control of public R-loop datasets has further highlighted that mapping modality can materially shape the inferred R-loop landscape, reinforcing the need for careful method selection and orthogonal validation when telomeric R-loops are used as mechanistic intermediates [43]. The functional consequences of these epitranscriptomic mechanisms for telomere homeostasis and disease contexts are summarized in Fig. 2A.

### Disease and stress connections

Telomere maintenance readouts have been embedded in clinical practice and disease modeling, which has made the field unusually well suited for linking epitranscriptomic perturbations to medically interpretable endpoints. Telomere length distributions have been used to stratify telomere biology disorders and to distinguish disease from healthy aging, while telomerase activity assays have remained relevant in translational oncology and selected diagnostic contexts and to evaluation of telomerase-directed therapies [34, 37]. Because the epitranscriptome has been repeatedly integrated into DNA damage responses and stress signaling, telomere phenotypes observed after RNA modification perturbation have been recommended to be interpreted in parallel with stress readouts (replication stress, DNA damage signaling, and cell-cycle effects) rather than as isolated endpoints rather than as isolated endpoints [19, 42, 43] (Fig. 2B). In addition, the expansion of nanopore-based native RNA sequencing has been discussed as a potential bridge from mechanistic epitranscriptomics to clinically oriented measurement, provided that detection accuracy, input requirements, and analytical reproducibility are further improved [27, 28, 44].

## Epitranscriptomic regulation of the telomerase RNP

Telomerase-mediated telomere maintenance is executed by a ribonucleoprotein (RNP) in which the catalytic protein TERT is organized around the noncoding telomerase RNA TERC (hTR), together with accessory factors that govern RNP maturation, trafficking, and productive telomere engagement [3, 45]. In human cells, telomerase output has been found to depend strongly on post-transcriptional control of hTR, including the kinetics of RNA processing and the partitioning of hTR across nuclear compartments that either permit or restrict assembly with TERT [3, 45–47]. Recent work has further shown that the DBHS proteins NONO, SFPQ, and PSPC1 associate with hTR-containing telomerase, promote telomerase trafficking out of Cajal bodies, and facilitate telomerase recruitment to telomeres, reinforcing the importance of RNA-centered trafficking steps in productive telomerase function [48]. Because telomerase insufficiency underlies many telomere biology disorders (TBDs), and telomerase upregulation sustains most cancers, telomerase biogenesis has been positioned as both a mechanistic bottleneck and a therapeutic entry point [3, 45, 46, 49].

Within this framework, epitranscriptomic regulation of the telomerase RNP has been supported most directly by studies in which chemical marks on hTR, or enzymes that install/remove those marks, have been linked to telomerase assembly, activity, or recruitment [3, 22, 45, 50–53]. Two conceptually distinct layers have been emphasized: internal nucleotide modifications within hTR that can remodel local structure or protein binding, and specialized 5′ cap chemistry that can redirect RNA fate and RNP trafficking [3, 22, 45, 50–55]. These regulatory layers are schematically summarized in Fig. 1.

### Internal RNA modifications that tune hTR–protein assembly

A site-specific cytosine methylation event on hTR has been connected to telomerase RNP formation. Methylation at hTR C106 (m⁵C) has been shown to be promoted by the RNA-binding protein HuR, likely by facilitating access of an as-yet-unidentified m⁵C methyltransferase, and enhanced C106 methylation has been associated with improved assembly of the hTR–TERT complex, increased telomerase activity, and maintenance of telomere length [22, 47]. Conversely, multiple disease-associated hTR variants have been reported to impair HuR–hTR association, reduce C106 methylation, diminish telomerase activity, and accelerate telomere shortening, thereby providing a direct route by which an RNA modification axis can be coupled to a clinically recognizable telomere maintenance defect [22].

An additional internal mark has been implicated through m⁶A-dependent regulation. Endogenous hTR has been reported to carry m⁶A, and the m⁶A demethylase ALKBH5 has been validated as an hTR-interacting factor capable of erasing this modification on cellular hTR [50]. Altered ALKBH5 levels have been associated with changes in telomerase complex assembly and telomerase enzymatic activity, consistent with a model in which the m⁶A state of hTR influences productive RNP formation [50]. More recent work has strengthened this model by mapping m⁶A sites on TERC at A111 and A435, identifying METTL3 as the writer, and defining YTHDC1 as a scaffold that promotes TERT–TERC assembly and telomerase function, thereby moving this pathway from a broadly plausible model toward a more site-resolved mechanism [56]. This mechanism has been of particular interest because it links a reversible and drug-adjacent RNA mark to a core step in telomerase regulation that is otherwise difficult to manipulate with specificity [3, 50].

### hTR 5′ cap hypermethylation as a telomerase routing signal

hTR is transcribed by RNA polymerase II and is therefore born with a 7-methylguanosine (m⁷G) cap that can be further remodeled into a 2,2,7-trimethylguanosine (TMG) cap by the hypermethylase TGS1 [3, 45]. In human cells, loss of TGS1 has been shown to increase steady-state hTR abundance, increase assembled telomerase levels, and promote telomere elongation, indicating that TGS1-dependent cap maturation can function as a negative constraint on telomerase biogenesis under some conditions [51]. Pharmacologic inhibition of TGS1 using the S-adenosylmethionine analog sinefungin has similarly been reported to raise hTR levels and promote telomere lengthening in multiple cell types, and a rationale has been proposed for leveraging this effect in TBD settings where hTR is limiting (noting that sinefungin is a broad SAM-dependent methyltransferase inhibitor and that cellular effects can reflect inhibition of multiple methyltransferases in addition to TGS1) [53]. Consistent with and extending this model, recent work has shown that TGS1-dependent 5′-cap trimethylation promotes decay of long genomically extended hTR precursors and functionally cooperates with PAPD5-dependent 3′ oligoadenylation to control hTR fate, with combined inhibition synergistically increasing hTR in cells carrying pathogenic telomerase mutations [57].

At the same time, TMG capping has been shown to carry information beyond bulk hTR abundance. In lung cancer cells and tumor organoids, TGS1-dependent TMG capping has been reported to be required for efficient telomerase recruitment to telomeres and for engagement of Cajal body–linked steps of telomere maintenance [52, 58]. In that context, TGS1 depletion or inhibition has been associated with Exo1-dependent generation of recombinogenic telomeric substrates, RAD51-dependent recombination, and activation of key ALT-like features, implying that cap state can bias selection between telomerase-dependent maintenance and recombination-based maintenance when telomere end structure is remodeled [52]. Taken together, the TGS1–TMG axis has been positioned as a telomerase biogenesis and trafficking module whose net phenotypic output can depend on cellular context, duration of perturbation, and the availability of backup telomere maintenance routes [51–53].

Evolutionary conservation of this principle has been supported in budding yeast, where Tgs1 has been shown to generate the m³G cap on the telomerase RNA TLC1 and where Tgs1 loss has been reported to alter telomere length and structure and shorten replicative lifespan, linking cap hypermethylation to chromosome-end homeostasis across eukaryotes [54]. Thus, cap-dependent control of telomerase RNA appears conserved at the level of principle, whereas the underlying RNA scaffolds and accessory-factor interactions are more clearly lineage-specific [12, 59]. Comparative analysis across fungi has likewise identified conserved core domains in telomerase RNAs despite extensive sequence divergence, while plant telomerase studies indicate that conserved accessory proteins can be deployed through lineage-specific RNA structural assemblies [12].

### Structural RNA chemistry and catalytic tuning

Evidence has also been provided that base modifications can subtly adjust telomerase catalytic behavior through structural effects on hTR domains that contact TERT. Pseudouridylation within the catalytically essential P6.1 hairpin has been shown to remodel local RNA loop structure and thermodynamic stability, and modest changes in telomerase activity and processivity have been reported in vitro when Ψ is incorporated at predicted sites [55]. Although the physiological installation and dynamics of these Ψ sites in vivo remain less well resolved than the m⁵C and cap-hypermethylation axes above, the observations have supported the broader premise that RNA chemistry can tune telomerase not only through abundance and recruitment, but also through catalytic efficiency [55].

### Disease and stress connections

A disease-facing link has been established most clearly for hTR m⁵C, where multiple TBD-associated hTR variants have been shown to disrupt HuR binding, reduce C106 methylation, and impair telomerase function in parallel with telomere shortening phenotypes [22]. For the TGS1 module, a translational opportunity has been suggested by the observation that sinefungin-mediated TGS1 inhibition can increase hTR levels and promote telomere lengthening, with explicit relevance proposed for stem and progenitor cells from TBD patients in whom hTR is reduced [53]. In cancer, the same pathway has been shown to influence telomerase recruitment and to permit ALT-like features when telomerase engagement is compromised, implying that epitranscriptomic control of hTR cap state could be exploited either to reinforce telomerase-dependent maintenance or to destabilize it in a way that exposes recombination dependencies [52]. Finally, the demonstration that ALKBH5 can erase m⁶A on hTR and modulate telomerase assembly has suggested that telomerase activity may be indirectly coupled to broader m⁶A network states that are frequently remodeled in tumors and stress-adapted cell programs [3, 50].

## Epitranscriptomic regulation of telomeric RNAs

Telomeres have been shown to be transcriptionally active, and mammalian chromosome ends have been demonstrated to produce long noncoding transcripts termed telomeric repeat–containing RNA (TERRA) that are heterogeneous in size, arise from multiple subtelomeric loci, and localize to telomeres [8]. Telomeric transcription has been attributed primarily to RNA polymerase II, and telomeric RNAs have been described as UUAGGG-repeat–containing products whose abundance has been reported to vary with developmental state, telomere length, tumor type, cellular stress, and chromatin configuration [9]. A recent synthesis has further emphasized that TERRA biogenesis is evolutionarily conserved across eukaryotes and that TERRA functions may extend beyond telomere maintenance itself [60]. In recent syntheses, TERRA has been positioned as a multifunctional regulator operating at the intersection of telomere damage signaling, telomere rescue, and pathway choice between semiconservative replication and recombination-based maintenance [61]. At the same time, telomere-to-telomere differences in TERRA production and handling have been emphasized, implying that telomeric RNA regulation cannot be treated as a purely global property of the nucleus [7]. Beyond mammals, telomere-specific regulation of TERRA has also been demonstrated in *Caenorhabditis elegans*, where POT-1 and POT-2 repress TERRA expression in a chromosome-end-specific manner, further supporting the idea that TERRA regulation is evolutionarily conserved but mechanistically species-specific [62].

### RNA partitioning and movement as determinants of telomeric function

A mechanistic leverage point has been placed in the partitioning of TERRA between telomere-bound and “free” pools, because telomere-associated TERRA has been argued to influence local telomeric transactions (chromatin configuration, replication progression, and telomerase access), whereas extratelomeric TERRA has been considered a reservoir that can be redistributed under changing telomere states [7, 61]. Dynamic redistribution has been directly supported by imaging-based evidence in human cells in which TERRA has been shown to colocalize with the telomerase RNA subunit hTR in the nucleoplasm and at telomeres, and telomeric TERRA has been reported to act in trans to inhibit telomere elongation by telomerase [63]. Consistent with this inhibitory logic, biochemical work showed that TERRA can bind human telomerase and directly inhibit telomerase activity in vitro [64]. In that work, relocation of newly transcribed TERRA away from its chromosome end of origin toward long telomeres was documented, and depletion of TERRA was shown to promote telomeric localization and residence time of hTR, consistent with a model in which telomerase access is constrained by TERRA occupancy at chromosome ends [63]. In other contexts, telomere-shortening–induced TERRA has been proposed to nucleate telomerase molecules and promote their recruitment to short telomeres, underscoring that TERRA–telomerase coupling can be context- and telomere-state dependent [65]. These findings have reinforced that TERRA abundance alone is unlikely to be the decisive parameter; rather, the rules governing TERRA stability, compartmentalization, and telomere targeting have been implicated as central determinants of phenotype [7, 63].

### m⁶A writing and reading stabilize *TERRA* and shape telomere-proximal RNA fate

In mammalian systems, direct epitranscriptomic regulation of TERRA has been established through N⁶-methyladenosine (m⁶A). m⁶A has been detected on TERRA and has been mapped predominantly to subtelomeric segments of the transcript, and its installation has been attributed to METTL3 [23]. Recognition of m⁶A-modified TERRA by the nuclear reader YTHDC1 has been shown to stabilize TERRA, and knockdown of either METTL3 or YTHDC1 has been reported to accelerate TERRA degradation and shorten TERRA half-life [23]. Importantly, a causal contribution of site-specific methylation to TERRA stability has been supported using targeted perturbation: site-directed demethylation of a mapped m⁶A site in a defined TERRA transcript (via dCas13–ALKBH5) has been shown to reduce local m⁶A signal and to lower the abundance of the targeted TERRA species [23]. Collectively, these results have indicated that telomeric RNA stability can be tuned by an RNA mark–reader axis, rather than being dictated solely by transcriptional output from subtelomeric promoters (Fig. 1).

### m⁶A-dependent telomere targeting and coupling to chromatin-interacting RNA behavior

Beyond bulk stabilization, telomere targeting of TERRA has been linked to m⁶A status in ALT-positive settings. Evidence has been presented that m⁶A within TERRA is enriched in R-loop–associated TERRA fractions, and recruitment of hnRNPA2B1 to TERRA has been reported to require m⁶A and to be necessary for efficient telomeric targeting through R-loop–dependent association [24]. Consistent with a functional requirement, loss of METTL3 or disruption of TERRA m⁶A has been associated with telomere damage in ALT-positive cells, and pharmacologic METTL3 inhibition has been reported to compromise telomere targeting of TERRA and to increase telomeric DNA damage in ALT-positive neuroblastoma models [24]. Although telomeric R-loops are discussed in depth elsewhere (Sect. 5), the telomeric RNA–specific implication is that epitranscriptomic marks can operate as “routing information” for a chromatin-interacting lncRNA, with telomere localization and retention being influenced by chemical state rather than sequence alone [24].

### Telomere factors as modulators of TERRA-linked methylation dynamics

Epitranscriptomic control of telomeric RNA has also been suggested to be coupled to telomere-binding proteins that coordinate RNA methylation turnover. The telomeric zinc finger protein ZBTB48 (TZAP) has been shown to associate with the m⁶A/m⁶Am demethylase FTO and to bind both mRNAs and the telomere-associated RNA TERRA, and ZBTB48 depletion has been reported to reduce targeting of FTO to methylated sites, alter cellular m⁶A/m⁶Am levels, and change decay rates of FTO-regulated transcripts [66]. While the direct consequences for TERRA methylation stoichiometry remain to be defined in a site-resolved manner, the findings have provided a plausible mechanism by which a telomere factor could coordinate demethylase access to telomere-associated RNA substrates and thereby modulate RNA stability programs linked to telomere maintenance [66].

### Disease and stress connections

Telomeric transcription has been repeatedly connected to stress contexts, and TERRA regulation has been framed as a telomere-proximal stress response rather than a constitutive housekeeping process [7, 9, 61]. In vivo evidence has been provided that TERRA levels can rise in human blood under extreme environmental conditions (spaceflight and high-altitude climbing), and hybridized TERRA at telomere-specific double-strand breaks and accumulation of TERRA foci in G2-phase have been directly visualized following induction of telomeric double-strand breaks in ALT cells [67]. These observations have supported the view that telomeric RNA pools are remodelled during stress and damage, thereby creating a context in which RNA stabilization or destabilization by m⁶A writer/reader/eraser systems could materially influence telomere outcomes [23, 24, 66, 67]. Finally, the demonstration that METTL3 inhibition can induce telomeric DNA damage in ALT-positive tumor models has suggested that telomeric RNA methylation may be exploitable therapeutically, provided that ALT selectivity and systemic liabilities of m⁶A pathway modulation are carefully resolved [24].

## Epitranscriptomics at telomeric R-loops and genome stability

Telomeric RNA: DNA hybrids (R-loops) have been positioned as a functional intermediate that can couple telomeric transcription to chromosome-end maintenance, while also creating a liability for replication and genome stability [68–70]. In budding yeast lacking telomerase, TERRA accumulation has been shown to occur preferentially at the shortest telomeres, where persistent telomeric R-loops have been linked to DNA damage signaling and RAD51 recruitment, thereby promoting homology-directed repair (HDR) events that slow senescence onset [68]. In human ALT cells, telomeric RNA: DNA hybrids formed with TERRA have been shown to require tight control, because RNaseH1 depletion has been associated with excess hybrid accumulation, exposure of single-stranded telomeric DNA, telomeric RPA activation, and abrupt telomere excision, whereas RNaseH1 overexpression has been linked to reduced recombinogenicity and telomere shortening [69]. These observations have supported a “Goldilocks” model in which telomeric R-loops must be maintained within a productive range: sufficient to enable recombination-based transactions, yet limited enough to avoid catastrophic replication stress and telomere loss [68, 69]. Consistent with this dose- and context-dependent logic, telomeric RNA: DNA hybrids in budding yeast were shown to promote recombination-mediated telomere elongation in recombination-competent telomerase mutants, but to accelerate telomere loss and senescence when homologous recombination is unavailable [71]. Major regulators of telomeric R-loop formation and resolution, and their reported phenotypic consequences for telomere stability and pathway engagement, are summarized in Table 4.

**Table 4:** Telomeric R-loop homeostasis: regulators, directionality, and outcomes

Factor/pathway	Primary role in telomeric *R*-loop control	Phenotype when perturbed	Implication for TMM	Key References
RNaseH1	Hybrid removal (resolution)	Depletion: excess hybrids, ssDNA exposure, telomere excision; Overexpression: reduced recombination, shortening	Goldilocks hybrid range for ALT maintenance	[69]
RAD51–BRCA2	Promotes TERRA targeting in trans; supports R-loop formation	Reduced targeting/hybrids when impaired	Enables recombination-leaning telomere transactions	[70]
TRF1	Antagonizes telomeric R-loops	Increased R-loop propensity when counteraction lost	Telomere replication protection	[70]
ATRX	Suppresses R-loops at transcribed telomeric repeats	Loss increases telomeric R-loops	Links chromatin remodeling defects to ALT-like contexts	[72]
BRCA1	Suppresses telomeric R-loop–driven damage; binds TERRA/shelterin	Loss: ↑TERRA, ↑telomeric R-loops, replication stress	Bridges tumor suppressor loss to telomere instability	[73]
RTEL1	Coordinates TERRA abundance/localization and telomeric hybrid state	Depletion: ↑TERRA but ↓TERRA-containing telomeric R-loops; RNaseH1 OE partially phenocopies	Telomere disorder linkage; hybrid balance	[74]
C19orf43 (RNA-binding factor)	Represses persistent telomeric R-loops affecting sister telomere cohesion	Loss: reduced damage and delayed senescence in aged cells	R-loops as structural elements in mitosis/aging	[76]
Induced TERRA transcription	Drives R-loop formation experimentally	Triggers BIR and PRIMPOL-dependent repair; replication interference	Switch toward recombination-like repair dependencies	[77]
METTL3–YTHDC1 (m⁶A on TERRA)	Stabilizes TERRA and supports hybrid-mediated ALT outputs	METTL3/YTHDC1 loss: reduced R-loops, telomere instability	Epitranscriptomic “hybrid competence” layer	[23]
hnRNPA2B1 (m⁶A-dependent on TERRA)	Promotes R-loop formation and telomere targeting	Disruption: telomeric damage; loss of targeting	Therapeutic angle in ALT neuroblastoma	[24]
ADAR1p110 (A-to-I editing)	Edits mismatches in hybrids at variant repeats to enable resolution	Editing loss: hybrid persistence/genome instability; proliferation defect	Editing dependency under telomere sequence heterogeneity	[80]

Network view of telomeric R-loop formation and removal, integrating core hybrid enzymes with telomere and repair factors, plus epitranscriptomic inputs that tune hybrid competence

### Formation and turnover of telomeric R-loops: cis, trans, and cell-cycle control

Telomeric R-loops have been shown to form not only co-transcriptionally at the chromosome end of origin but also post-transcriptionally in trans, thereby allowing telomeric RNA to be redistributed to other chromosome ends [70]. A RAD51-dependent pathway has been demonstrated in which UUAGGG repeat RNA has been found to be sufficient for telomere targeting, and telomere association and R-loop formation have been shown to be promoted by RAD51 and BRCA2 while being counteracted by RNaseH1 and TRF1 [70]. A telomere length bias has also been documented: short telomeres have been shown to accumulate TERRA and R-loops because of local defects in RNA degradation and RNase H–dependent hybrid processing, and persistence of these hybrids has been linked to altered coordination between TERRA turnover and telomere replication [68].

Multiple pathways have been implicated in restraining telomeric R-loops at transcribed telomeric repeats. ATRX recruitment has been shown to depend on repeat transcription and other repeat features, and loss of ATRX has been associated with increased R-loop formation at telomeric repeats, consistent with a role for ATRX in suppressing deleterious secondary structures that arise in transcribed telomeric DNA [72]. A complementary control point has been defined for BRCA1: direct binding of BRCA1 to TERRA and telomeric shelterin proteins has been shown to occur in an R-loop- and cell-cycle–dependent manner, and BRCA1 loss has been associated with upregulated TERRA expression, overly abundant telomeric R-loops, and telomeric replication stress [73]. In addition, RTEL1 has been shown to influence both TERRA abundance and its localization, with RTEL1 depletion being associated with elevated TERRA levels but reduced TERRA-containing R-loops at telomeres, and RNaseH1 overexpression has been reported to partially phenocopy RTEL1 deficiency [74]. Together, telomeric R-loop homeostasis has been indicated to be enforced by a network that integrates RNA surveillance, recombination factors, and chromatin-associated suppressors.

### Genome stability consequences: replication stress, cohesion, and repair pathway engagement

Telomeric R-loops have been shown to interfere with semiconservative DNA replication under conditions where they accumulate inappropriately, thereby generating telomere fragility and repair-associated telomere damage [70, 73]. Recent work further suggests that telomeric RNA: DNA hybrids can also support replication restart under selected conditions, as TRF1-mediated suppression of telomere fragility was found to depend on fork reversal and RNA: DNA hybrids in human cells [75]. In yeast lacking telomerase, persistent hybrids at short telomeres have been linked to DDR activation and recruitment of recombination machinery, implying that “damage-like” signaling at telomeres can be harnessed for telomere rescue when telomerase is absent [68]. In human cells, a protective telomere-end use case has been described in which TERRA R-loops have been shown to hold sister telomeres together in mitosis; repression of these hybrids has been linked to the RNA-binding protein C19orf43, and relief of persistent cohesion by RNaseH1 has supported a direct structural role for telomeric hybrids in sister telomere cohesion [76]. In aged cells, depletion of C19orf43 has been reported to reduce DNA damage and delay replicative senescence, consistent with controlled telomeric R-loop formation being able to buffer telomere attrition–associated stress [76].

At the opposite extreme, telomeric R-loops have been shown to become toxic when replication and repair demand are forced beyond capacity. A recent causal test has been provided in which TERRA transcription and telomeric R-loop formation were experimentally induced in telomerase-expressing cells, and telomeric R-loops were found to be sufficient to interfere with semiconservative replication and to trigger engagement of break-induced replication (BIR) and PRIMPOL-dependent repriming [77]. Synthetic lethality between PRIMPOL depletion and BIR deficiency has further suggested that telomeric R-loop–driven replication interference can create parallel repair dependencies that resemble those exploited by ALT cells [77]. These findings have indicated that telomeric R-loops can function as a switch-like input into repair pathway choice, depending on magnitude, timing, and the availability of compensatory synthesis routes [68, 69, 77].

### Epitranscriptomic marking of telomeric hybrids: m6A as a tunable “hybrid competence” layer

In mammalian cells, direct epitranscriptomic control of telomeric R-loops has been established most clearly through m6A on TERRA. m6A has been reported to occur on subtelomeric regions of TERRA, to be deposited by METTL3, and to be read by YTHDC1, with stabilization of TERRA being linked to telomere protection and telomere maintenance outputs in ALT settings [23]. R-loop reduction, telomere shortening, and telomere instability have been reported upon METTL3 depletion, consistent with m6A-dependent TERRA stability being coupled to a hybrid-mediated telomere maintenance mechanism [23]. A mechanistic extension has been reported in which m6A has been found to be abundant in R-loop–enriched TERRA fractions, and m6A-mediated recruitment of hnRNPA2B1 has been shown to be critical for R-loop formation and telomere targeting of TERRA, with telomeric DNA damage being induced upon disruption of this axis [24]. Pharmacologic METTL3 inhibition has also been reported to compromise telomere targeting of TERRA and increase telomeric DNA damage in ALT-positive neuroblastoma models, thereby reinforcing that telomeric hybrid control can be drug-responsive at the level of an RNA modification pathway [24].

A broader principle has been supported by genome-wide DNA damage studies in which m6A pathway components have been shown to shape DNA: RNA hybrid dynamics at DSBs. METTL3 activation and localization to DNA damage sites have been reported to promote m6A installation on damage-associated RNAs, and YTHDC1-dependent protection of these RNAs has been linked to DNA: RNA hybrid accumulation that recruits HR factors and supports DSB repair [78]. In a complementary model, ARID1A-dependent recruitment of METTL3/METTL14 to R-loops has been reported to install m6A on R-loop RNA and to facilitate RNase H1 recruitment, thereby promoting R-loop resolution and genome stability [79]. Although these DSB-centric mechanisms are not telomere-exclusive, they have provided experimentally tractable templates by which telomeric m6A-marked hybrids could be stabilized or resolved in a context-dependent manner, rather than being treated as passive by-products of transcription at chromosome ends [23, 24, 78, 79].

### A-to-I editing as telomere-specific R-loop quality control

A telomere-restricted form of R-loop “proofreading” has been reported via ADAR1-mediated A-to-I RNA editing. In cancer cells carrying non-canonical variant telomeric repeats, the nuclear ADAR1p110 isoform has been shown to edit A–C mismatches within RNA: DNA hybrids formed between canonical and variant repeats, and conversion of mismatches to I: C pairs has been shown to facilitate RNase H2–mediated resolution of telomeric R-loops [80]. Continued proliferation of telomerase-reactivated cancer cells with variant repeats has been reported to require this ADAR1p110-dependent control of telomeric R-loops, thereby linking an epitranscriptomic reaction (A-to-I editing) directly to telomeric genome stability in a genetically defined telomere context [80].

### Disease and stress connections

Disease-relevant connections have been embedded in several telomeric R-loop control points. Recent work has also shown that oxidative stress can induce TERRA upregulation, cis- and trans-acting telomeric RNA: DNA hybrids, TRF1 dissociation, and TRF2-dependent R-loop formation, providing a mechanistic link between redox stress and telomeric RNA remodeling [81]. RTEL1 mutations have been implicated in telomere biology disorders, and RTEL1 deficiency has been shown to disrupt TERRA abundance/localization and to produce telomeric instability that can be partially phenocopied by experimental hybrid removal [74]. BRCA1 loss or mutation has been linked to excess TERRA-associated R-loops and telomeric replication stress, thereby connecting a canonical tumor suppressor pathway to telomere-centered genome instability through hybrid control [73]. In telomerase-reactivated cancer cells bearing telomeric variant repeats, dependence on ADAR1p110 has been attributed to a requirement for editing-enabled telomeric R-loop resolution, suggesting that telomere sequence heterogeneity can create an “editing dependency” under proliferative stress [80]. Finally, the ability of induced telomeric R-loops to trigger BIR and PRIMPOL-dependent repair has suggested that stressors elevating telomeric transcription or hybrid persistence could bias cells toward repair programs that are normally constrained at healthy telomeres, with potential implications for both aging-associated telomere fragility and ALT-associated cancer vulnerabilities [68, 76, 77].

## Epitranscriptomic control of ALT and telomere maintenance networks

Alternative lengthening of telomeres (ALT) has been defined as a telomerase-independent telomere maintenance mechanism in which telomere extension is achieved through homology-directed repair and long-range DNA synthesis, frequently described within a break-induced replication (BIR)-like framework [82, 83]. Although telomerase remains dominant across cancers, ALT has been repeatedly emphasized as a clinically meaningful minority program because distinct nuclear organization, repair dependencies, and therapeutic liabilities have been associated with ALT-positive tumors [82, 84].

### ALT as a condensate-centered maintenance program

ALT-associated promyelocytic leukemia (PML) bodies (APBs) have been positioned as organizing hubs where telomeres are clustered and repair factors are concentrated to support telomeric DNA synthesis [82, 84]. Telomere clustering has been reconstituted in polySUMO/polySIM nuclear condensates and has been linked to mitotic DNA synthesis programs that engage BLM and RAD52, thereby connecting phase separation principles to ALT-like telomere synthesis [85]. APBs have also been shown to behave as liquid condensates in response to telomeric DNA damage, with condensation and coalescence proposed to facilitate both factor enrichment and telomere–telomere encounters [86]. Beyond canonical PML scaffolding, SUMO-driven condensate behavior has been suggested to remain operative: SUMO-dependent recruitment and collaboration among repair proteins have been reported to support ALT-like telomere maintenance even in the absence of PML [87]. Consistent with this logic, telomeric SUMO levels have been shown to bias APB formation pathways and to modulate ALT efficiency and telomeric DNA synthesis output [88].

### Telomeric RNAs as scaffolds and signals within ALT networks

A prominent conceptual shift has been created by the placement of telomeric RNAs—particularly TERRA and telomeric RNA: DNA hybrids (telR-loops)—as functional components of ALT rather than passive transcriptional by-products [89]. Recent work has further linked ALT maintenance to RNA-centered telomere regulation by showing that TOP3A stabilizes shelterin in ALT cells and is required for TERRA enrichment at telomeres, thereby extending the set of factors that couple telomeric RNA handling to ALT telomere architecture [90]. Within this view, telomeric RNA has been treated as a potential scaffold for the selective concentration of proteins in telomeric condensates. A biophysical example has been provided in which TERRA binding to LSD1 has been shown to promote phase separation, enrich R-loop–stimulating factors, and increase TERRA-containing telomeric hybrids in a manner required for ALT cell fitness [91]. In parallel, a direct epitranscriptomic entry point has been established through m6A on TERRA: METTL3-dependent m6A has been linked to TERRA stability and telomere stability in ALT settings, and m6A-dependent telomere targeting of TERRA has been coupled to recombination-associated telomere maintenance and telomeric damage phenotypes when the pathway is disrupted [23, 24].

### RNA modifications as modulators of condensate behavior and pathway choice

A mechanistic bridge between RNA methylation and condensate assembly has been supported outside the telomere field by evidence that mRNAs carrying multiple m⁶A marks can act as multivalent scaffolds that enhance phase separation of YTHDF proteins and alter RNA partitioning into phase-separated compartments [92]. m6A-dependent phase separation behavior has also been demonstrated for YTHDF2, reinforcing that reader–RNA binding can influence droplet formation and material properties [93]. When these findings are considered alongside the condensate-centered architecture of ALT and the requirement for m6A-marked TERRA in ALT maintenance, a plausible model is created in which epitranscriptomic marks on telomeric RNAs could influence the recruitment, retention, or physical state of telomeric maintenance condensates [23, 24, 85–88, 92, 93]. Direct demonstration of such “material-property” control at telomeres has remained limited, and stringent separation of telomere-proximal effects from global m6A network perturbation has therefore been required for causal claims [82, 84, 92, 93].

### Epitranscriptomic rewiring of telomere-factor networks

Telomere maintenance state has also been shown to be reshaped through RNA modification of transcripts encoding telomere regulators. HMBOX1/TAH1 has been identified as a telomere-associated homeobox protein that binds telomeric DNA, associates with PML bodies, and contributes to ALT phenotypes, including APB formation or persistence [94]. Importantly, *HMBOX1* mRNA has been established as a functional m6A target in cancer: METTL3-catalyzed methylation has been reported to promote HMBOX1 mRNA loss via an m6A-reader–dependent decay route, leading to defective telomerase recruitment to telomeres, cumulative telomere shortening, telomere dysfunction, and chromosomal instability, with reversibility shown by HMBOX1 reintroduction or targeted m6A manipulation [95]. In this way, epitranscriptomic remodeling has been connected to telomerase recruitment capacity and to genome instability—two pressures that can plausibly influence whether telomerase-based maintenance is sustained or whether recombination-based alternatives become selectively advantageous [82, 95]. These pathway-level outcomes and their disease relevance are summarized in Fig. 2.

A reciprocal direction of control has also been suggested: a telomeric zinc finger protein (ZBTB48) has been shown to recruit the demethylase FTO to RNA targets and to bind both mRNAs and TERRA, indicating that telomere-associated proteins can steer epitranscriptomic enzyme access to selected RNA substrates [66]. Whether this axis imposes telomere-length–dependent control over specific telomere maintenance pathways remains unresolved, but a framework has been created in which telomere proteins and the epitranscriptome can be mutually regulatory [66].

### Disease and stress connections

ALT has been enriched in specific tumor lineages and has been framed as a therapeutic opportunity because ALT-specific nuclear bodies and repair dependencies have been repeatedly documented [82, 84]. Within this context, disruption of METTL3-dependent *TERRA* methylation has been connected to impaired telomere targeting of *TERRA* and increased telomeric damage in ALT-positive neuroblastoma models, supporting the feasibility of targeting an RNA-modification axis to destabilize ALT telomere maintenance [24]. In telomerase-positive cancers, a distinct vulnerability has been suggested by the METTL3–HMBOX1 axis, where telomere dysfunction and genome instability have been tied to an epitranscriptomic program that impairs telomerase recruitment rather than telomerase catalysis itself [95]. Environmental and physiological stress have also been implicated as amplifiers of these links: under toxicant exposure, m6A remodeling has been reported to affect Hmbox1 stability and telomere length phenotypes in reproductive injury models, illustrating that telomere–epitranscriptome coupling can be revealed in stress-conditioned settings [96], and that telomere dynamics may intersect with environmental cue–responsive signaling pathways controlling pace-of-life traits via reproductive timing (e.g., Hippo/YAP/TAZ) [97].

## Conclusion and outlook

A shift has been underway in how telomere maintenance is conceptualized: control has not been confined to telomeric DNA, shelterin, and chromatin, but has also been distributed across RNA substrates and RNA-centered genome stability pathways. In parallel, RNA modifications have been increasingly integrated into DNA damage response logic, where RNA marks and RNA: DNA hybrids have been positioned as active determinants of repair efficiency and stress signaling, rather than passive by-products of transcription [19]. Within telomere biology, several mechanistic exemplars have now made “epitranscriptomic control” difficult to dismiss as circumstantial. Telomerase output has been shown to be modulated by an m⁵C-dependent axis on TERC that reshapes telomerase RNP assembly and has been perturbed by telomeropathy-linked TERC variants [22]. A second routing layer has been provided by 5′ cap hypermethylation of hTR: TGS1-dependent TMG capping has been linked to telomerase recruitment and has been implicated in restricting a switch toward recombination-driven maintenance under conditions that otherwise favor ALT-like features [52]. On the telomeric transcript side, m⁶A-dependent targeting of TERRA to telomeres has been coupled to hybrid formation and ALT-associated maintenance, and telomere damage has been induced when this pathway has been disrupted in ALT models [24]. A further telomere-proximal quality-control principle has been illustrated by A-to-I editing: ADAR1p110 has been shown to edit mismatched bases within telomeric RNA: DNA hybrids formed on variant repeats, facilitating RNase H2–mediated resolution and supporting proliferation of telomerase-reactivated cancer cells [80]. Beyond telomeric RNAs themselves, a network-level route has been demonstrated in which m⁶A-mediated destabilization of HMBOX1 mRNA has been linked to impaired telomerase recruitment, progressive telomere shortening, and telomere dysfunction in cancer cells [95].

A unifying interpretation has been suggested by these findings: RNA marks at chromosome ends may function as (i) routing codes that bias RNA localization and RNP engagement, (ii) hybrid competence signals that tune whether telomeric RNA: DNA hybrids are formed, stabilized, or resolved, and (iii) network rewiring inputs that reshape the telomere maintenance state indirectly through modified mRNA fate. Across taxa, conservation is clearest at the level of RNA-dependent telomerase biogenesis, telomeric transcript regulation, and hybrid homeostasis, whereas direct evidence for individual telomere-associated RNA modification pathways remains more lineage-restricted. A particular opportunity has been created by the growing view of ALT as a condensate-organized maintenance program, because multivalent RNA–protein interactions and material properties of nuclear bodies could plausibly be modulated by RNA chemical state, thereby offering a physical mechanism for epitranscriptomic influence over pathway choice and telomere synthesis efficiency [82]. A more systematic comparative synthesis across taxa will be an important next step for the field, particularly as telomere-relevant RNA regulation becomes more deeply resolved outside mammalian systems.

Several experimental priorities appear to be decisive for the next phase of the field. First, causal inference should be strengthened by site-directed manipulation of specific marks, rather than reliance on global enzyme perturbation, which remains vulnerable to pleiotropy and secondary stress phenotypes; CRISPR-guided RNA editing platforms have already provided a workable route for such tests [31]. Second, stoichiometry and heterogeneity of modifications on telomere-relevant RNAs should be resolved more directly, because low-occupancy marks could still be decisive if they are concentrated in the telomere-bound fraction; native RNA sequencing has been positioned as one path toward concurrent quantification of isoforms and modifications, although clinical-grade robustness remains an active challenge [27]. Third, temporal resolution should be treated as essential: telomere phenotypes frequently accumulate over many divisions, whereas RNA marks can be remodeled on short timescales during stress, replication conflicts, or DNA damage, creating a mismatch that can confound interpretation unless kinetics are explicitly tracked [98].

Translational implications have also been sharpened, but have remained context-dependent. In short-telomere settings where hTR abundance is limiting, pharmacologic inhibition of TGS1 has been reported to increase hTR levels and promote telomere lengthening, suggesting a plausible disease-oriented strategy that is mechanistically distinct from direct telomerase activation [53]. Conversely, in ALT-positive cancers, disruption of TERRA methylation and targeting has been proposed as a vulnerability, but systemic liabilities of broadly perturbing m⁶A circuitry will need to be separated from telomere-specific dependencies. Finally, biomarker strategies are likely to benefit from higher-resolution telomere phenotyping: long-read telomere measurement has enabled telomere-length distribution features to be linked to healthy aging and telomere biology disorders, and this resolution may be required to detect modest but clinically meaningful shifts produced by epitranscriptomic interventions [37]. Potential therapeutic entry points and key caveats for targeting RNA-modification pathways in telomere maintenance are summarized in Table 5.

**Table 5:** Translational opportunities and caveats for targeting RNA-modification axes in telomere maintenance

Target axis	Intervention concept	Expected telomere effect	Best-fit context	Major caveats	Key references
TGS1 → hTR TMG cap	Inhibit TGS1 (e.g., sinefungin as tool compound)	↑hTR levels; may promote elongation; affects recruitment and precursor decay	Short-telomere/TBD-like settings; also cancer context	Sinefungin is a broad SAM-inhibitor; PAPD5-linked precursor processing may contribute; TMM switching/ALT-like features possible	[51–53, 57]
METTL3 → m⁶A on TERRA	METTL3 inhibition	Reduced telomere targeting of TERRA; increased telomeric damage	ALT-positive tumors (e.g., neuroblastoma models)	Systemic m⁶A liabilities; need ALT selectivity	[23, 24]
ALKBH5 ↔ hTR m⁶A	Modulate ALKBH5 activity/levels	Alter telomerase assembly/activity	Telomerase-driven cancers (hypothesis-driven)	Sites/stoichiometry on hTR not fully resolved; pleiotropy	[3, 50]
ADAR1p110 editing at variant repeats	Target editing dependency	Destabilize telomerase-reactivated cells with variant repeats	Subset of cancers with telomeric variant repeats	Broad roles of ADAR1; stratification required	[80]
m⁶A decay of HMBOX1 mRNA	Interrupt methylation/reader-mediated decay	Restore telomerase recruitment competence; reduce instability	Telomerase-positive cancers where axis is active	Need tumor-context validation; global m⁶A impacts	[95]

Candidate intervention points with mechanistic rationale, disease context, and key caveats (off-target liabilities, context dependence, compensatory TMM switching)

## Data Availability

No datasets were generated or analysed during the current study.

## Abbreviations

ALT: Alternative lengthening of telomeres
APB: ALT-associated PML body
A-to-I: Adenosine-to-inosine RNA editing
ATRX: Alpha thalassemia/mental retardation syndrome X-linked chromatin remodeler
BIR: Break-induced replication
BRCA1: Breast cancer type 1 susceptibility protein
BRCA2: Breast cancer type 2 susceptibility protein
C-circles: Extrachromosomal telomeric C-rich circles (ALT marker)
C19orf43: RNA-binding factor implicated in telomeric R-loop repression and sister telomere cohesion
Cas13: CRISPR-associated RNA-guided RNase used for RNA targeting/editing platforms
dCas9: Nuclease-dead Cas9 used for programmable targeting without DNA cleavage
dCas13: Nuclease-dead Cas13 used for programmable RNA targeting without RNA cleavage
DDR: DNA damage response
DEHP: Di(2-ethylhexyl) phthalate
DSB: Double-strand break
FFPE: Formalin-fixed paraffin-embedded
Flow-FISH: Flow cytometry–fluorescence in situ hybridization
FTO: Fat mass and obesity-associated protein (m⁶A/m⁶Am demethylase)
HDR: Homology-directed repair
HMBOX1 (TAH1): Homeobox containing 1, telomere-associated homeobox protein
hnRNPA2B1: Heterogeneous nuclear ribonucleoprotein A2/B1
hTR: Human telomerase RNA (TERC)
HuR (ELAVL1): Hu antigen R RNA-binding protein
m⁶A: N⁶-methyladenosine
m⁶Am: N⁶,2′-O-dimethyladenosine (cap-adjacent)
m⁵C: 5-methylcytosine
m⁷G: 7-methylguanosine (canonical Pol II RNA cap)
m³G: 2,2,7-trimethylguanosine cap
METTL3: Methyltransferase-like 3
METTL14: Methyltransferase-like 14
PAMmer: Short DNA oligonucleotide enabling RNA targeting by Cas9-based editors
PML: Promyelocytic leukemia protein
PRIMPOL: Primase–polymerase
Pol II: RNA polymerase II
Q-FISH: Quantitative fluorescence in situ hybridization
qPCR: Quantitative polymerase chain reaction
RAD51: Recombinase RAD51
R-loop: RNA: DNA hybrid with displaced single-stranded DNA
RNase H1: Ribonuclease H1
RNase H2: Ribonuclease H2
RPA: Replication protein A
RNP: Ribonucleoprotein
SAM: S-adenosylmethionine
SIM: SUMO-interacting motif
S9.6: RNA: DNA hybrid-detecting antibody
TeSLA: Telomere shortest length assay
TERC: Telomerase RNA component
TERT: Telomerase reverse transcriptase
TERRA: Telomeric repeat-containing RNA
TBD: Telomere biology disorder
TIF: Telomere dysfunction-induced foci
TMG: 2,2,7-trimethylguanosine cap
TMM: Telomere maintenance mechanism
TRAP: Telomeric repeat amplification protocol
TRF: Terminal restriction fragment (assay) and telomeric repeat-binding factor (context-dependent)
TRF1: Telomeric repeat-binding factor 1
TRF2: Telomeric repeat-binding factor 2
TGS1: Trimethylguanosine synthase 1
TZAP (ZBTB48): Telomeric zinc finger-associated protein
YTHDC1: YTH domain-containing protein 1
YTHDF2: YTH N6-methyladenosine RNA binding protein 2
Ψ: Pseudouridine
